# The Surgical Treatment of Osteoarthritis

**DOI:** 10.3390/life12070982

**Published:** 2022-06-30

**Authors:** Peter Brumat, Ožbej Kunšič, Samo Novak, Urban Slokar, Janez Pšenica, Matevž Topolovec, Rene Mihalič, Rihard Trebše

**Affiliations:** 1 Valdoltra Orthopaedic Hospital, 6280 Ankaran, Slovenia; samo.novak@ob-valdoltra.si (S.N.); urban.slokar@ob-valdoltra.si (U.S.); matevz.topolovec@ob-valdoltra.si (M.T.); rene.mihalic@ob-valdoltra.si (R.M.); rihard.trebse@ob-valdoltra.si (R.T.); 2 Faculty of Medicine, University of Ljubljana, 1000 Ljubljana, Slovenia; ozbej.kunsic@sb-je.si; 3 Division of Surgery, Department of Traumatology, General Hospital Jesenice, 4270 Jesenice, Slovenia; jani.psenica@sb-je.si; 4 Faculty of Medicine, University of Maribor, 2000 Maribor, Slovenia

**Keywords:** osteoarthritis, spine, hip, knee, ankle and foot, shoulder, elbow, wrist and hand, computer-assisted orthopaedic surgery, CAOS

## Abstract

Osteoarthritis is a degenerative condition affecting the whole joint with the underlying bone, representing a major source of pain, disability, and socioeconomic cost worldwide. Age is considered the strongest risk factor, albeit abnormal biomechanics, morphology, congenital abnormality, deformity, malalignment, limb-length discrepancy, lifestyle, and injury may further increase the risk of the development and progression of osteoarthritis as well. Pain and loss of function are the main clinical features that lead to treatment. Although early manifestations of osteoarthritis are amenable to lifestyle modification, adequate pain management, and physical therapy, disease advancement frequently requires surgical treatment. The symptomatic progression of osteoarthritis with radiographical confirmation can be addressed either with arthroscopic interventions, (joint) preservation techniques, or bone fusion procedures, whereas (joint) replacement is preferentially reserved for severe and end-stage disease. The surgical treatment aims at alleviating pain and disability while restoring native biomechanics. Miscellaneous surgical techniques for addressing osteoarthritis exist. Advanced computer-integrated surgical concepts allow for patient personalization and optimization of surgical treatment. The scope of this article is to present an overview of the fundamentals of conventional surgical treatment options for osteoarthritis of the human skeleton, with emphasis on arthroscopy, preservation, arthrodesis, and replacement. Contemporary computer-assisted orthopaedic surgery concepts are further elucidated.

## 1. Background

Osteoarthritis is a degenerative condition affecting the whole joint with the underlying bone, representing a major source of pain, disability, and socioeconomic cost worldwide [1]. It is thought to be the most prevalent chronic joint disease [2]. The epidemiology of the disorder is complex and multifactorial, with genetic, biological, and biomechanical components [1]. Age is considered the strongest risk factor for osteoarthritis, albeit abnormal biomechanics, morphology, congenital abnormality, deformity, malalignment, limb-length discrepancy, lifestyle, and injury may further increase the risk of the development and progression of osteoarthritis as well [1,2,3,4].

Pain and loss of function are the main clinical features that lead to treatment, including non-pharmacological, pharmacological, and surgical approaches [2]. Although early manifestations of osteoarthritis are amenable to lifestyle modification, adequate pain management, and physical therapy, disease advancement frequently requires surgical treatment [1,4]. The symptomatic progression of osteoarthritis with radiographical confirmation can therefore be addressed either with arthroscopic interventions, joint preservation techniques, or bone fusion procedures, whereas joint replacement is preferentially reserved for severe and end-stage disease [1]. Surgical treatment aims at alleviating pain and disability while restoring native biomechanics. Miscellaneous surgical techniques for addressing osteoarthritis exist. Furthermore, advanced computer-integrated surgical concepts allow for patient personalization and optimization of surgical treatment. Three-dimensional printing has become more frequently used in surgical specialties in recent years, including pre-operative planning, patient-specific instrumentation, and patient-specific implant production [5]. The computer-assisted navigation system is another well-known orthopaedic advancement that allows the surgeon to obtain real-time feedback during surgeries [6]. Robotic technologies mainly aim at supporting surgeons with precise and planned mechanical actions, while technologies such as augmented reality increase the ability of the surgeon by intuitive augmentation of medical information [7].

The scope of this article is to present an overview of the fundamentals of conventional surgical treatment options for osteoarthritis of the human skeleton, with emphasis on arthroscopy interventions, (joint) preservation techniques, bone fusion (arthrodesis) concepts, and (joint) replacement methods. Contemporary computer-assisted orthopaedic surgery concepts are further elucidated. 

## 2. Arthroscopy

The adoption of arthroscopy, using small cameras and instruments placed inside joints, has transformed musculoskeletal care over the last several decades, allowing surgeons to provide the same anatomic solutions with less tissue dissection, resulting in lower requirements for inpatient care, reduced costs, and expedited recovery [8].

### 2.1. Spine

Despite numerous endoscopic methods that can be utilized in the treatment of cervical, thoracic, and lumbar degenerative spine disorders, they are most commonly performed through the posterolateral (or interlaminar) and the extraforaminal (or transforaminal) approach [8]. The three most commonly utilized techniques include full endoscopy, microendoscopy, and biportal endoscopy, each with its characteristics [8]. 

### 2.2. Shoulder

If osteoarthritis of grade II–III is present, arthroscopic debridement and microfractures are the treatments of choice in young adults [9]. A more advanced procedure is comprehensive arthroscopic management (CAM) [10]. The arthroscopic debridement and interposition of an acellular human dermal matrix for glenoid resurfacing can also be performed in carefully selected patients [11]. In more advanced or extended unipolar/bipolar osteoarthritis, arthroscopic-assisted partial shoulder resurfacing and arthroscopic biologic total shoulder resurfacing remain options [12,13].

### 2.3. Elbow

For advanced osteoarthritis of the elbow, refractory to non-operative measures can be addressed arthroscopically. It is considered when the joint surface is preserved, and the main problem is a reduced range of movement caused by osteophytes, intra articular loose bodies, and tight articular capsules [14]. 

### 2.4. Wrist and Hand

Wrist arthroscopy for OA includes the debridement of osteophytes, synovectomy, distal radial styloidectomy, as well as proximal row carpectomy (PRC) or arthroscopically assisted partial fusion for advanced disease [15,16,17]. At the level of the distal radioulnar joint (DRUJ), the removal of loose bodies and capsulorrhaphy is an option in the early stages, while arthroscopic resection of the ulnar head (Wafer procedure) or the arthroscopic Sauve–Kapandji procedure are options for advanced disease [15,16]. The thumb carpometacarpal joint osteoarthritis can be addressed arthroscopically with thermal stabilization of the joint capsule, or arthroscopic (hemi-)trapeziectomy [18].

### 2.5. Hip

Hip arthroscopy allows detailed visualization of the acetabular labrum, femoral and acetabular chondral surfaces, fovea, ligamentum teres, synovium, and the extra-articular peri-trochanteric space [19]. Thus, any pathology of these structures that may consequentially lead to osteoarthritis can be addressed via arthroscopy [19]. Debridement alone, a microfracture of the exposed bone, foreign body removal, chondrogenic procedures, cartilage repair, autologous chondrocyte implantation techniques, and autologous matrix-induced chondrogenesis can be performed [19].

### 2.6. Knee

Osteochondral defects can predispose to early-onset osteoarthritis in young patients, and given the right indication, an all-arthroscopic autologous chondrocyte implantation (ACI) or arthroscopic mosaicplasty can be performed [20,21], as well as all-autologous matrix-induced chondrogenesis (AMIC), or hyaluronan-based scaffold implantation on chondral defects in dry arthroscopy [22]. Despite concerns regarding its efficacy, arthroscopic debridement remains a good option for short-term relief of symptoms in selected patients with knee osteoarthritis [23]. Incisionless nanoscopy for partial meniscectomy has also been reported recently [24]. 

### 2.7. Ankle and Foot

Arthroscopic debridement and microfractures of the ankle form a safe procedure with good to excellent reported outcomes [25]. Cartilage defects can also be treated with the transplantation of the hyaline cartilage (auto/allograft) or allograft cartilage matrix [26,27]. Arthroscopy of the first metatarsophalangeal (MTP) joint has reported a high satisfaction rate for the treatment of the initial stages of first MTP joint osteoarthritis (hallux rigidus) [28] 

## 3. Preservation

With increasing life expectancy, there is a growing demand for the preservation of native articular cartilage to delay or prevent osteoarthritis onset or the progression of symptomatic degeneration and the eventual need for joint arthroplasty, especially in younger and active patients [29]. An alternative to endoscopic-assisted preservation surgery and arthroplasty may therefore present periarticular or corrective, biomechanics restoring, osteotomy, whereas miscellaneous implantable devices also exist as an alternative for a fusion. Appropriate patient selection and careful preoperative planning are vital for optimizing outcomes [30].

### 3.1. Spine

**Figure 1:**
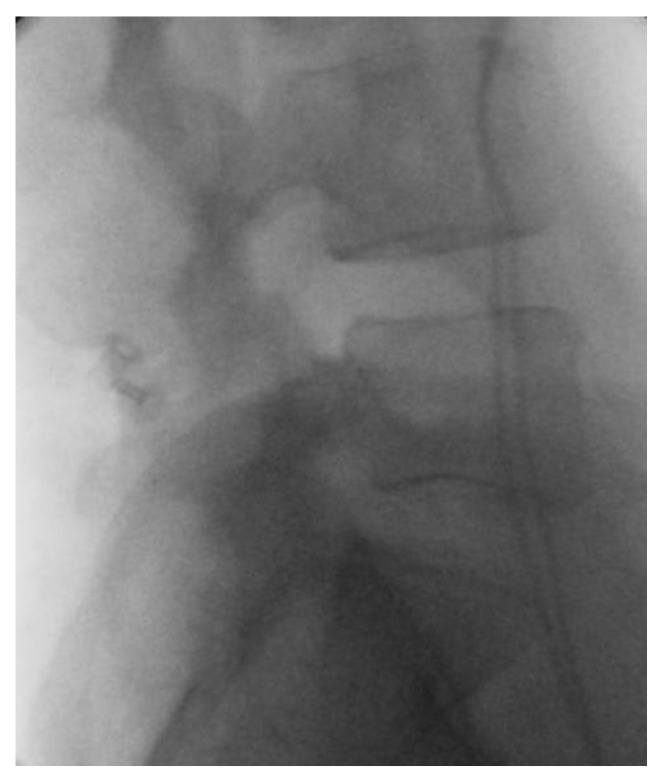
Interspinous spacer. Postoperative X-ray after interspinous spacer implantation (L4–L5 segment).

The primary goal of motion preservation surgery in the spine is to maintain normal or near-normal motion in an attempt to prevent adverse outcomes commonly seen with conventional spinal fusion, most notably the development of adjacent segment degeneration and disease [31]. Several different surgical approaches have been developed to preserve motion in the spine, including disc replacement, interspinous spacers (Figure 1), dynamic stabilization devices, and total facet replacement devices [31].

### 3.2. Shoulder

Osteotomies around the shoulder may be used in selected cases as a way of preventing or delaying the degeneration associated with morphologically and/or biomechanically abnormal articulation [32,33,34,35].

### 3.3. Elbow

**Figure 2:**
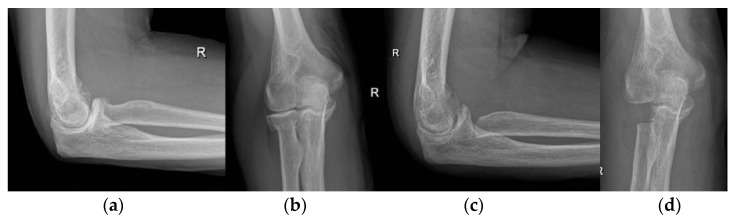
Interposition (anconeus) arthroplasty. (**a**,**b**) preoperative X-rays; (**c**,**d**) postoperative X-rays after interposition anconeus arthroplasty.

**Figure 3:**
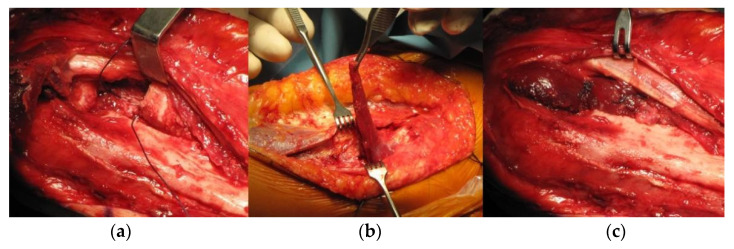
Interposition (anconeus) arthroplasty, intraoperative images. (**a**) radial head resection; (**b**) anconeus muscle preparation; (**c**) the final position of the interposed anconeus muscle.

For elbow osteoarthritis, distraction interposition arthroplasty uses grafts like fascia, tendon, or skin to create a new joint surface without the need for the resection of the destroyed joint. Osteo-capsular arthroplasty consisting of capsular releases and osteophyte removal, either performed openly or arthroscopically, is another feasible surgical option in young and active patients [36]. In radiocapitellar cartilage destruction, the resection of the radial head can be combined with the interposition of anconeus (Figure 2 and Figure 3) or with radiocapitellar implant arthroplasty [37,38].

### 3.4. Wrist and Hand

Total wrist denervation is a symptomatic treatment for selected patients with midcarpal osteoarthritis. Radial styloidectomy, excision of proximal or distal pole of the scaphoid, and proximal row carpectomy (PRC) are all options for carpal OA of different etiologies [15]. Corrective osteotomy of the distal radius, which aims at the management of radiocarpal OA after malunion can be supported with patient-specific 3D planning and implants [39]. For end-stage DRUJ osteoarthritis, many resectional procedures are described, albeit none of them without associated complications. These are partial distal ulnar resection (Wafer procedure), hemiresection interposition arthroplasty (Bowman’s procedure), distal ulnar resection (Darrach procedure), and the Sauve–Kapandji procedure, which is the arthrodesis of DRUJ with resection osteotomy of the distal ulna shaft [40]. Most common joint preserving procedures in thumb carpometacarpal joint osteoarthritis are denervation and metacarpal extension osteotomy [18].

### 3.5. Hip

**Figure 4:**
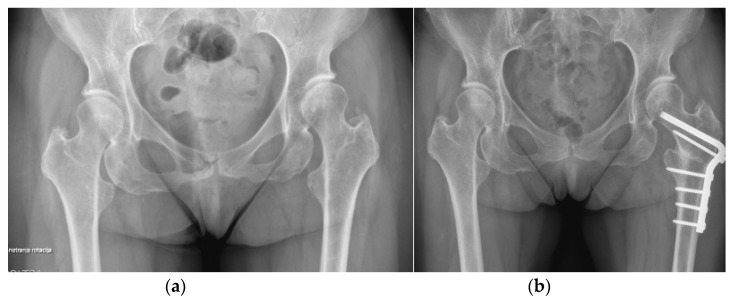
Varus femoral osteotomy. (**a**) preoperative X-ray; (**b**) postoperative X-ray.

**Figure 5:**
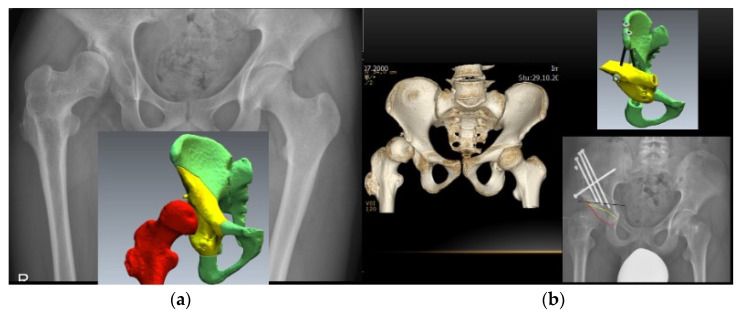
Periacetabular osteotomy (PAO). (**a**) preoperative X-ray and patient-specific 3D preoperative planning; (**b**) preoperative 3D CT, planned position of the 3D reoriented acetabular fragment and postoperative X-ray after PAO according to the preoperative patient-specific 3D plan.

Typical indications for hip-preserving surgery are: femoroacetabular impingement (intra- and extra-articular) (FAI), hip dysplasia, slipped capital femoral epiphysis (SCFE), residual deformities after Perthes disease, and avascular necrosis (AVN) of the femoral head [41]. If left unaddressed, these pathologies may lead to osteoarthritis. An unaddressed leg-length discrepancy may lead to osteoarthritis of the hip as well [42]. FAI and labral damage can be treated either with arthroscopy, surgical hip dislocation, and/or acetabular or femoral osteotomy [41]. Nowadays, severe or unstable SCFE can be treated open using surgical hip dislocation with the development of a retinacular soft tissue flap to perform a subcapital realignment of the slipped epiphysis, the so-called ‘modified Dunn’ procedure [41]. Residual deformities after Perthes disease can be treated with a semi-circumferential femoral osteochondroplasty, whereas a femoral head reduction osteotomy can be considered in selective cases if the containment of the femoral head cannot be achieved with femoral osteochondroplasty alone [41]. Depending on the localization, extension, and stage of the AVN, multiple therapeutic options are available (rotational osteotomies, bone grafting, core decompression, and varus/flexion femoral osteotomy (Figure 4)) [41]. Despite various acetabular osteotomies that have been described for the correction of hip dysplasia, the Bernese periacetabular osteotomy (PAO) (Figure 5), which enables corrections in a tri-dimensional fashion, produces inherent stability of the acetabular fragment due to the polygonal cuts and furthermore the preservation of the posterior column, presents the standard of care nowadays [41].

### 3.6. Knee

**Figure 6:**
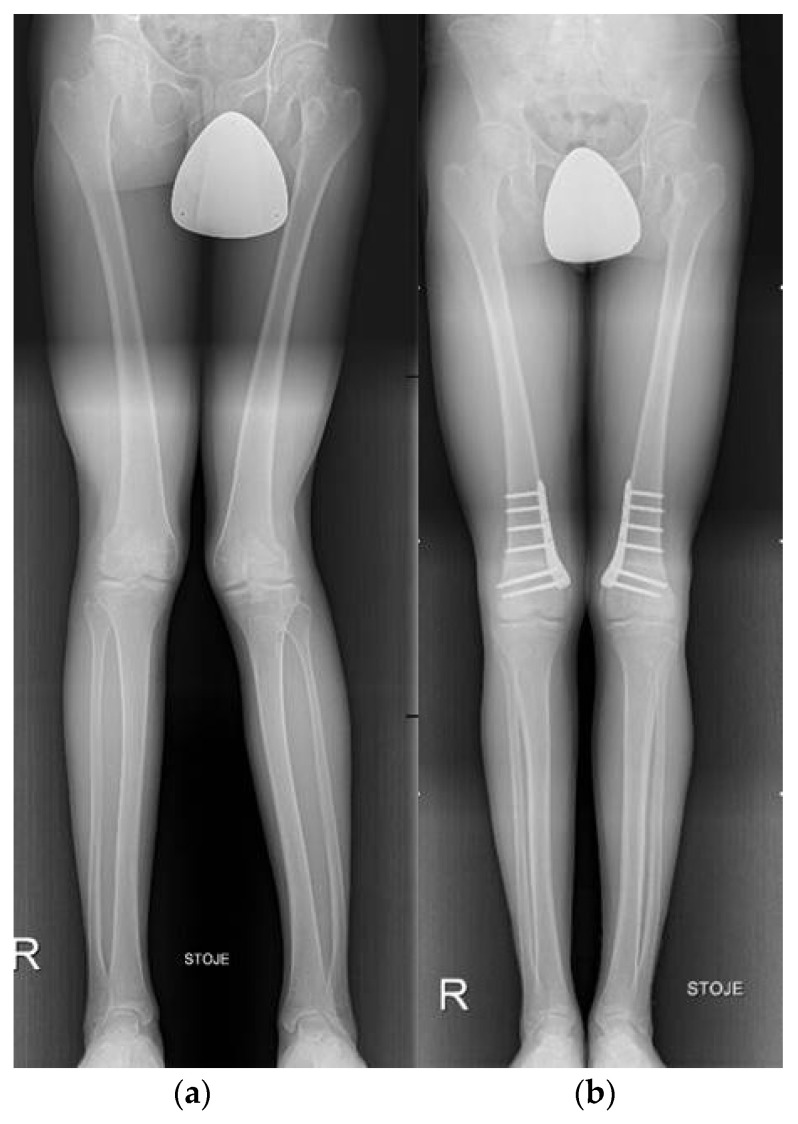
Bilateral distal femoral osteotomy (DFO). (**a**) preoperative X-ray of bilateral limb torsional deformity; (**b**) postoperative X-ray after bilateral DFO.

Indicated for the symptomatic single-compartment osteoarthritis and/or ligamentous instability of the knee, osteotomies around the knee are well-established surgical procedures which aim to decrease the load on the affected knee compartment by correcting the mechanical axis of the leg into a more optimal biomechanical position and more evenly distribute the forces across the joint surface [30,43,44]. Further advancement of osteoarthritis may thus be prevented. The two most common types of knee osteotomies are high tibial osteotomy (HTO) and distal femoral osteotomy (DFO) (Figure 6). In cases of severe knee malalignment, a combination of both can be performed with the aim of maintaining neutral joint-line obliquity and preserving the limb length [45].

### 3.7. Ankle and Foot

**Figure 7:**
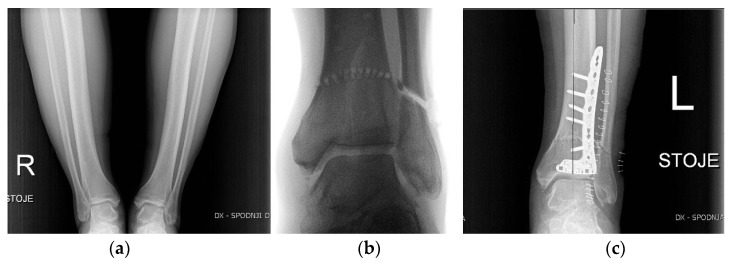
Dome-shaped supra-malleolar osteotomy. (**a**) preoperative X-ray; (**b**) intraoperative X-ray; (**c**) postoperative X-ray.

The most commonly performed ankle preservation procedures are supra-malleolar osteotomies (SMO) (Figure 7), indicated for patients with varus or valgus hindfoot deformity. The main cause of such deformities and concomitant asymmetric joint degeneration is post-traumatic instability of the joint [46]. Depending on the level of deformity, type of fixation, soft tissue covering, and joint presentation (congruent/incongruent), different types of osteotomies can be used [47]. Alternatively, technically more demanding multiplanar dome-shaped osteotomy (Figure 7) may preserve the congruity of the joint better than wedge osteotomies [48]. In some cases, complementary soft tissue balancing procedures or length correction of the fibula is suggested [49]. In patients with mild first MTP joint osteoarthritis, operative preservation procedures focus on removing excess osteophytes (cheilectomy) to prevent dorsal impingement with or without a concomitant osteotomy (Moberg) to improve or shift the range of motion into a less painful arc [50].

## 4. Arthrodesis

The induction of heterotopic osteosynthesis requires a complex balance of biological factors and operative technique with careful preparation of the fusion site and the appropriate selection of graft materials to achieve successful fusion, respecting the anatomical considerations including blood supply, osteology, and biomechanics which predispose a fusion site to robust or insufficient bone formation [51]. 

### 4.1. Spine

**Figure 8:**
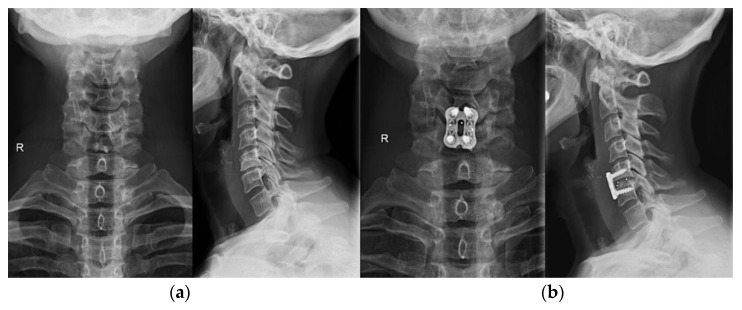
Anterior cervical discectomy and fusion (ACDF). (**a**) preoperative X-ray; (**b**) postoperative X-ray of the ACDF (C5–C6 segment).

Modern techniques of graft site preparation and instrumentation have evolved for every segment of the spine, with the aim of instrumentation to share loads with the host and graft tissues for the duration of the healing and remodeling phases [51]. Each construct is designed to avert the failure load of the implant or the bone-implant interface and resist applied forces with appropriate stiffness [51]. Fixation can be performed through percutaneous and minimally invasive approaches in selected cases. Although current technology in anterior cervical plating utilizes newer dynamic plate designs that maintain inter-segmental fixation and force distribution with the controlled collapse of a bone graft or strut in anterior cervical discectomy and fusion (ACDF) (Figure 8), the procedures have made placement easier and interbody fusion more successful; multilevel procedures without plating may remain subject to graft failure that can cause axial or angular collapse and nonunion [51]. Furthermore, lateral mass and pedicular screw fixation are possible methods for cervical arthrodesis, and the transarticular screws used alone or in combination with a rod can be used in occipito-cervical and C1-C2 constructs [51]. Instrumentation with posterior pedicle screw-rod fixation nowadays represents the standard of care combined with miscellaneous types of lumbar interbody fusion techniques and implants available, especially in unstable segments where interbody implants increase the chance of bone integration [51,52]. Polyetheretherketone (PEEK) and titanium (Ti) are commonly selected for interbody spacer construction [52]. The surface modification of Ti by creating rougher surfaces, modifying its surface topography (macro and nano), physical and chemical treatment, and creating a porous material with high interconnectivity, can improve its osseointegrative potential and bioactivity [52].

### 4.2. Shoulder

**Figure 9:**
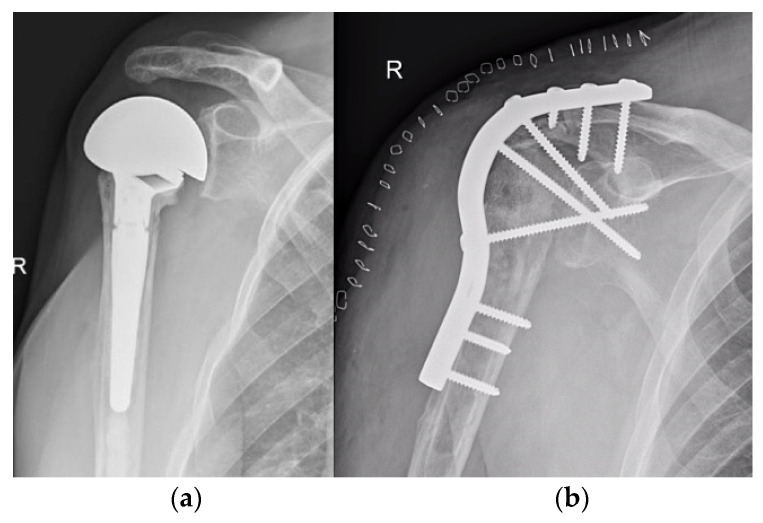
Shoulder arthrodesis. (**a**) preoperative X-ray after failed shoulder arthroplasty; (**b**) postoperative X-ray after shoulder arthrodesis.

Open or arthroscopic-assisted shoulder arthrodesis (Figure 9) is an end-stage procedure in selected cases of paralytic disorders, brachial plexus palsy, axillary nerve injuries, irreparable massive rotator cuff deficiency with arthropathy, after failed arthroplasty, proximal humerus tumor resection, inflammatory arthritis with rotator cuff pathology, and severe refractory instability, which involves fusion of the humeral head to the glenoid or to the acromion [53,54,55]. 

### 4.3. Elbow

**Figure 10:**
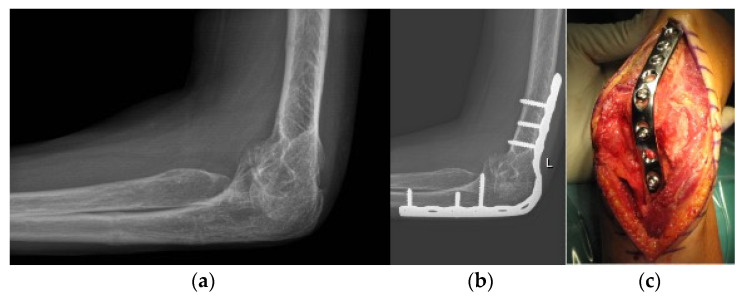
Elbow arthrodesis. (**a**) preoperative X-ray; (**b**) postoperative X-ray after elbow arthrodesis; (**c**) intraoperative image.

Elbow arthrodesis (Figure 10) is a rare salvage procedure with a highly functional deficit. The procedure is reserved for patients with highly unstable joints. Conversion to arthroplasty can be performed [56].

### 4.4. Wrist and Hand

In patients with localized wrist osteoarthritis, limited carpal fusion can be considered. For SLAC and SNAC wrists 4-corner arthrodesis is a well-established option, while scapho-trapezio-trapezoid (STT) fusion is indicated for isolated STT osteoarthritis. Less commonly performed is radio-scapho-lunate fusion. Total carpal fusion represents the ultimate salvage procedure in pancarpal arthrosis [15,57]. In a Sauve–Kapandji procedure, arthrodesis of the DRUJ is combined with ulnar shaft osteotomy [58]. CMC fusion of the thumb is an option for younger high-demand patients with advanced OA [18].

### 4.5. Hip

**Figure 11:**
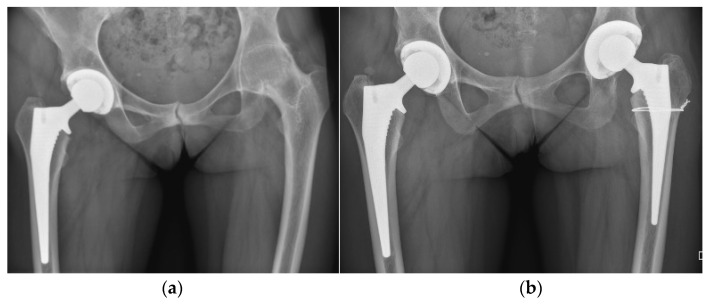
Secondary conversion of a hip arthrodesis to a total hip arthroplasty. (**a**) preoperative X-ray after left hip arthrodesis; (**b**) postoperative X-ray after secondary conversion to a total hip arthroplasty.

Arthrodesis used to be a treatment option for young adults or adolescents with unilateral hip disease, particularly in the presence of recent infection and especially in the setting of failed pelvic or hip surgery for trauma [59]. Proper patient selection and the optimal arthrodesis position were essential for a successful, long-term result; however, back and ipsilateral knee pain were the most common complaints leading to secondary conversion of a hip fusion to a total hip arthroplasty (Figure 11) [59].

### 4.6. Knee

**Figure 12:**
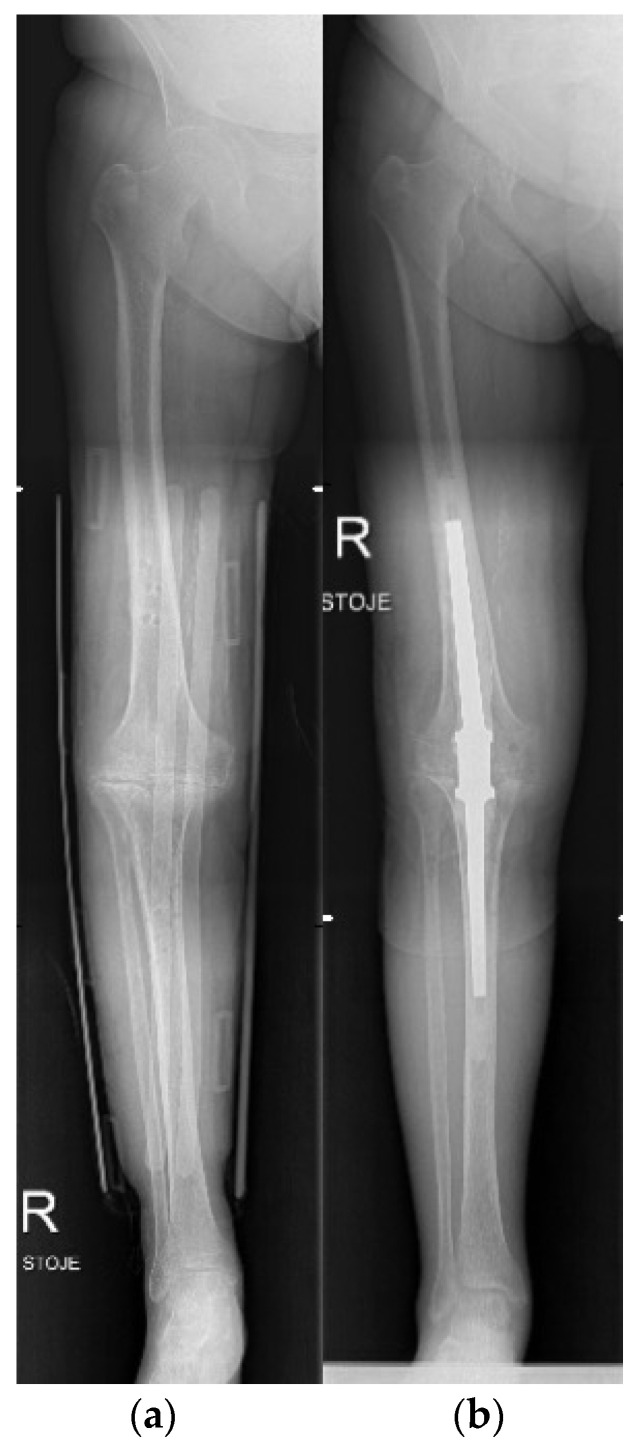
Knee arthrodesis. (**a**) preoperative X-ray in a splint; (**b**) postoperative X-ray after limb salvage knee arthrodesis with a nail.

**Figure 13:**
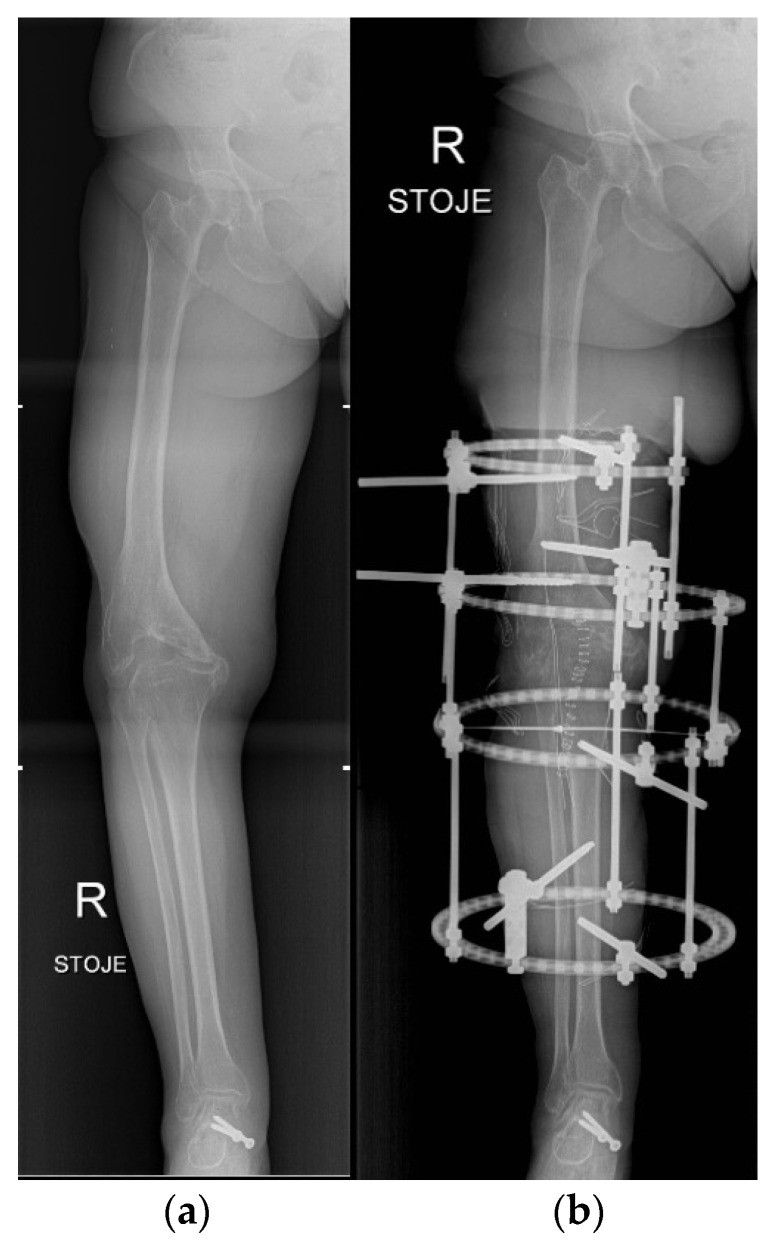
Knee arthrodesis. (**a**) preoperative X-ray of residual knee deformity after severe trauma; (**b**) postoperative X-ray after limb salvage knee arthrodesis with Ilizarov technique.

Knee arthrodesis (Figure 12) remains a limb salvage surgery for failed total knee arthroplasty, severe trauma, post/persistent infection, or tumor resection [60]. There are few studies describing techniques with limited indications without an extensive bone stock loss [61]. Alternatively, arthroscopic-assisted knee arthrodesis with the Ilizarov technique (Figure 13) is also reported [62]. 

### 4.7. Ankle and Foot

**Figure 14:**
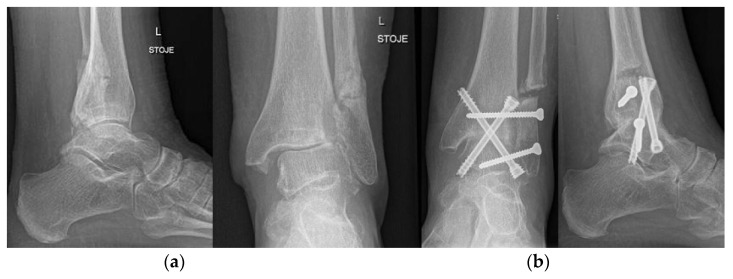
Ankle arthrodesis. (**a**) preoperative X-ray; (**b**) postoperative X-ray after ankle arthrodesis.

Ankle arthrodesis (Figure 14) presents a reliable treatment of end-stage ankle osteoarthritis with a reported 90% patients’ satisfaction, yielding better pain control after the procedure, faster rehabilitation, and being cheaper with shorter hospital stays [63,64,65,66]. Mostly, fusion is anterior tibiotalar, but can also be performed posteriorly and also for subtalar osteoarthritis [66,67]. Arthrodesis of the first MTP joint has demonstrated consistently good results in the literature and is the current standard of care for the treatment of advanced first MTP joint osteoarthritis, with numerous surgical techniques reported [50].

## 5. Replacement

Joint replacement is one of the most effective healthcare measures in improving patient quality-of-life outcomes, with a predicted future increase in the need for joint replacements as the population ages [68]. Both partial and total joint arthroplasty are nowadays well-established procedures in the scope of surgical osteoarthritis treatment. The primary purpose of all replacements is to alleviate pain while restoring the native articular surface and biomechanics. Miscellaneous combinations of component materials, designs, and sizes exist.

### 5.1. Spine

Cervical total disc arthroplasty is a safe and effective surgical alternative to arthrodesis in properly selected patients with cervical radiculopathy and myelopathy [69]. In carefully selected patients, lumbar disc replacement surgery has similarly been adopted as an alternative to fusion treatment for degenerative disc disease and may offer similar results to anterior-posterior fusion [70].

### 5.2. Shoulder

**Figure 15:**
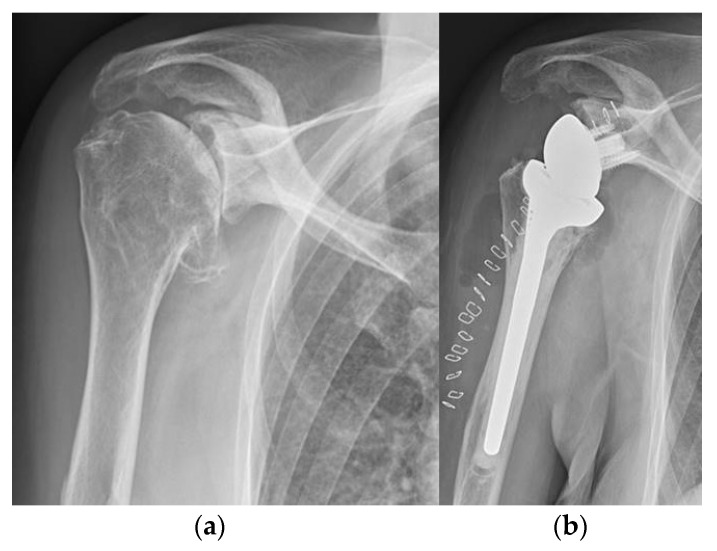
Reverse total shoulder arthroplasty (rTSA). (**a**) preoperative X-ray; (**b**) postoperative X-ray after reverse total shoulder arthroplasty.

Anatomic total shoulder arthroplasty (TSA) is considered a good long-term solution for end-stage glenohumeral osteoarthritis with an intact rotator cuff [71]. Shoulder hemiarthroplasty (HA) involves only the replacement of the humeral articular surface and is performed when glenoid resurfacing is contraindicated due to poor glenoid bone stock or in other selected cases [72,73]. Resurfacing hemiarthroplasty (RHA) has alternatively been proposed in an attempt to restore joint congruency while conserving proximal humeral bone stock and native anatomy of glenohumeral articulation [74]. Cuff-tear arthropathy of the shoulder has proved itself as a complex and difficult-to-treat form of shoulder degeneration associated with a massive chronic tear or other form of rotator cuff insufficiency that manifests as proximal migration of the humerus and erosion of the superior part of glenoid. The ensuing eccentric loading of the glenoid is also thought to be the main reason for early glenoid component loosening and the overall poor performance of the anatomic TSA used in this setting [75]. The treatment of cuff tear arthropathy with HA may provide adequate pain relief, but may not improve the functionality of the joint, especially elevation [76]. Reverse total shoulder arthroplasty (Figure 15) (rTSA) was therefore developed to address the limitations of preexisting prostheses with more anatomical design and is now considered an effective surgical treatment for end-stage osteoarthritis in rotator cuff deficient shoulders, as well as many other challenging shoulder pathologies [77].

### 5.3. Elbow

**Figure 16:**
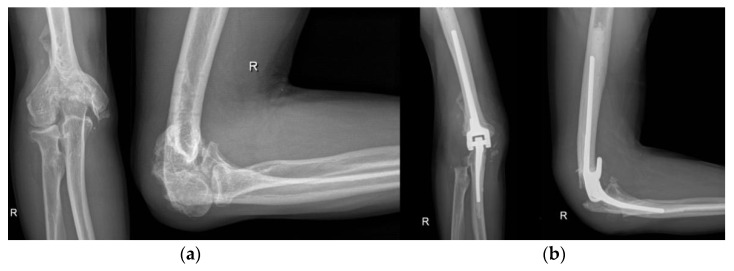
Total elbow arthroplasty. (**a**) preoperative X-ray; (**b**) postoperative X-ray after total elbow arthroplasty.

Total elbow arthroplasty (Figure 16) is surgical treatment for end-stage elbow osteoarthritis. Although was primarily indicated for rheumatoid arthritis of the elbow and posttraumatic derangements, is today a considerable option for treatment of end-stage primary osteoarthritis in selected patients, as the designs have evolved in recent decades [78]. 

### 5.4. Wrist and Hand

Total wrist arthroplasty is reserved for specific patients with end-stage wrist arthritis due to a lack of long-term survival, where the main concern remains periprosthetic osteolysis. For partial wrist replacement, treatments with implants that replace proximal carpal row are reported. Additionally, the pyrocarbon lunate resurfacing implants and pyrocarbon interposition implants are well-tolerated options for narrow indications [15,57]. Partial or total DRUJ arthroplasty with an implant is an alternative option for a destroyed joint [79]. For the OA of the fingers, numerous types of prosthetics are available. Current implants are designed of silicone, metal surface, or pyrolytic carbon, which can be fixed, semi-fixed or gliding [18].

### 5.5. Hip

**Figure 17:**
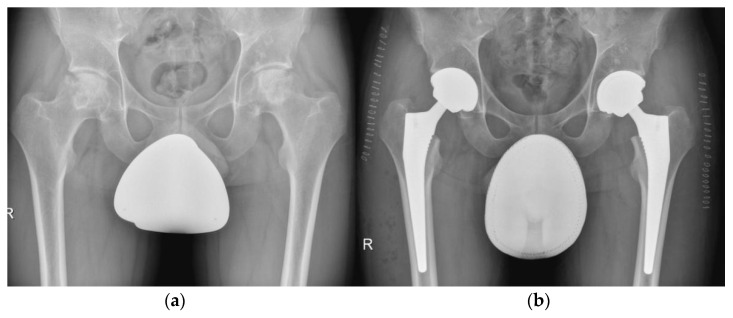
Bilateral total hip arthroplasty (THA). (**a**) preoperative X-ray; (**b**) immediate postoperative X-ray after one-stage bilateral total hip arthroplasty.

The cost-effectiveness of total hip arthroplasty (THA) (Figure 17) in treating advanced osteoarthritis makes it one of the most successful of all surgical interventions [80]. It has been described as “the operation of the 20th century” for the excellent results, the high satisfaction of the patients, and the improvement of the quality of life [81,82]. The reported hip prosthesis survival rate is as high as 51 years after implantation [83]. The components in a hip replacement consist of an acetabular cup, a femoral stem, and a head which articulates with the acetabular cup or a liner placed within the cup [84]. Larger heads confer stability and a greater arc of motion but create a greater volume of wear than smaller head sizes. The head can be created from stainless steel, cobalt-chrome or ceramic. The acetabular component may be composed of polyethylene or metal, or be metal backed, using a polyethylene or ceramic liner [84]. The cemented implants are placed into a bed of PMMA cement which secures the components into the prepared acetabulum and femoral canal, whereas uncemented implants have either a porous coating into which bone can grow or a roughened surface, produced by blasting the surface of the implant with microscopic particles to increase surface area for bone to grow onto [84]. Although different types of hip replacements composed of different materials exist, and the combination of ceramic-on-ceramic bearing is typically reserved for younger patients, metal and nowadays ceramic-on-polyethylene bearing continues to be the workhorse for the majority of cases [80,84]. The reported survival rates of THA are high even with mixed components [85]. Pelvic motion in spine-hip interaction can affect functional acetabular orientation, and consequently functional cup positioning in a THA may be recommended [86]. Proximal femoral morphology can also impact on THA outcome [87]. As an alternative to THA, and because of the theoretical benefits of femoral bone stock preservation, the hip resurfacing arthroplasty concept was developed for the treatment of young and active patients with hip osteoarthritis [88]. Dual mobility cup systems have gained increasing acceptance, especially in patients at high risk for dislocation [89]. Great advances have been introduced in the last few years in terms of less invasive surgical procedures, tissue preservation, improved wear resistance of the materials, biocompatibility and bone ingrowth capability of the biomaterials, knowledge and restoration of the hip anatomy, and function, peroperative management (pain control and blood loss), and prevention of complications [81]. 

### 5.6. Knee

**Figure 18:**
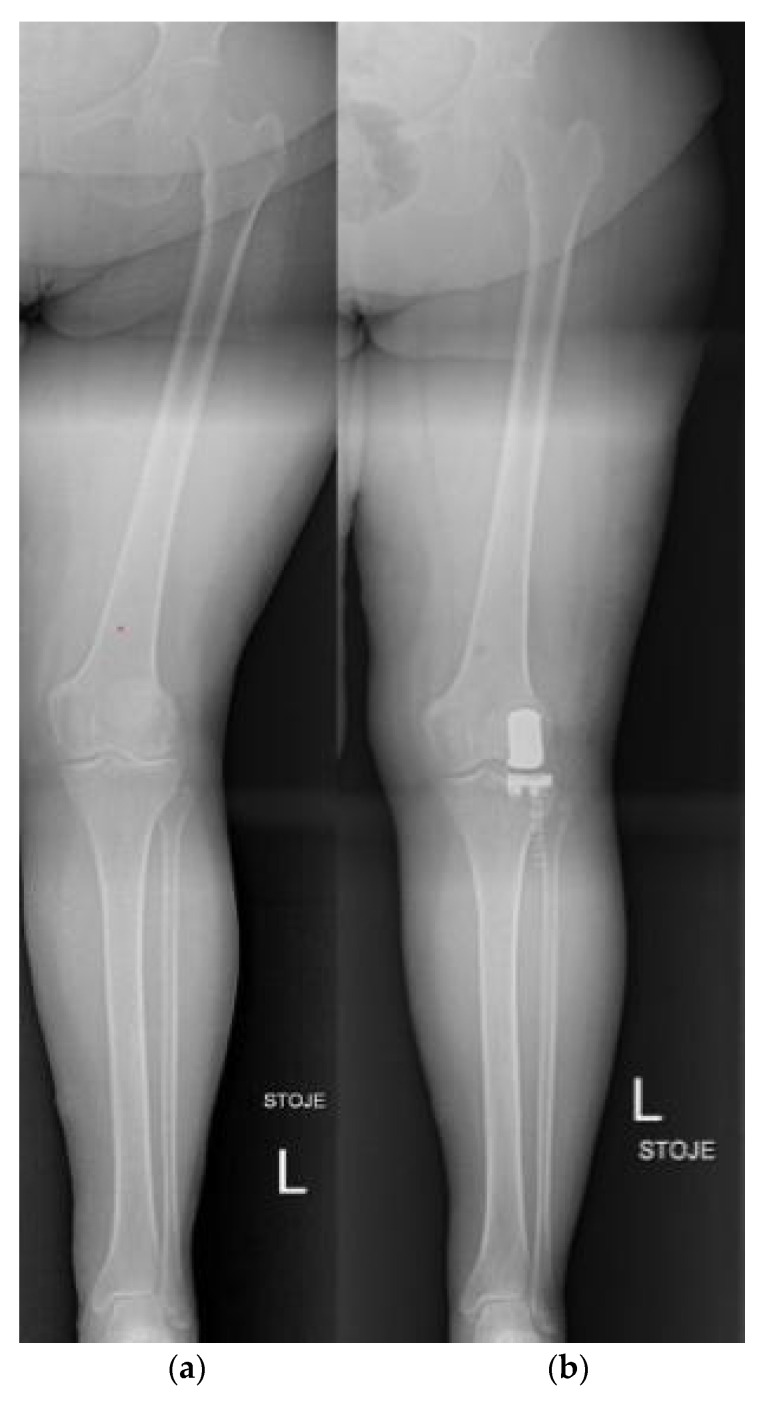
Unicompartmental (partial) knee arthroplasty (UKA). (**a**) preoperative X-ray; (**b**) postoperative X-ray after unicompartmental knee arthroplasty for isolated knee osteoarthritis of the lateral compartment.

**Figure 19:**
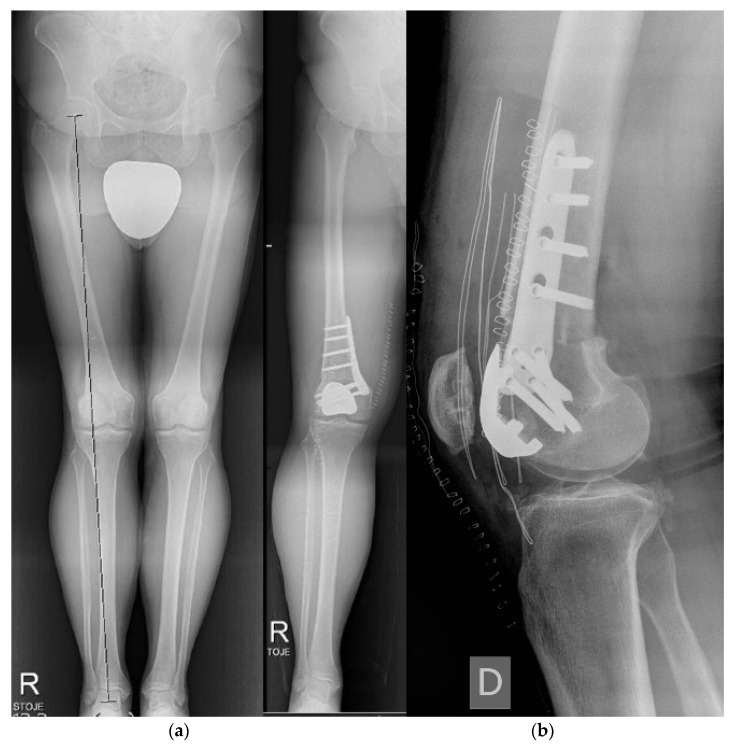
Patellofemoral arthroplasty. (**a**) preoperative X-ray of isolated patellofemoral osteoarthritis caused by torsional limb deformity; (**b**) postoperative X-ray after patellofemoral arthroplasty with concomitant distal femoral osteotomy (DFO) for limb deformity correction.

Total knee arthroplasty (TKA) represents one of the most cost-effective and reliable reconstructive surgical procedures in orthopaedics, used for the treatment of symptomatic end-stage primary knee osteoarthritis [90]. The cruciate-retaining TKA prosthesis design relies on the preserved posterior cruciate ligament (PCL) to provide stability in flexion. Beside retaining more natural knee kinematics, it also preserves more bone stock and native PCL proprioception [91]. The posterior-stabilized TKA design includes femoral cam that engages the tibial polyethylene post, which provides stability of the knee in flexion [92]. Mobile-bearing knee designs were developed as an alternative to fixed bearing implants to allow rotation (“rotating platform”) or translation (“meniscal bearing”) of the polyethylene insert on the tibial baseplate, in order to reduce wear of the bearing surfaces and improve implant survivorship [93]. Individuals with symptomatic osteoarthritis limited to either the medial or lateral compartment of the knee may benefit from unicompartmental (partial) knee arthroplasty (UKA) (Figure 18). These implants replace the articular surface of either the medial (more often) or lateral femoral condyle and the adjacent tibial plateau surface. In recent years, enthusiasm for UKA has been revived with the growing tendency toward minimally invasive surgery, although the literature suggests slightly worse survivorship in comparison to TKA [94]. For the sparing of the contralateral tibiofemoral and patellofemoral compartment with cruciate ligaments, UKA grants more natural knee kinematics, which also implies a faster postoperative rehabilitation time and better range of motion compared with TKA [95]. Patellofemoral arthroplasty (Figure 19) is also an option in isolated patellofemoral osteoarthritis.

### 5.7. Ankle and Foot

**Figure 20:**
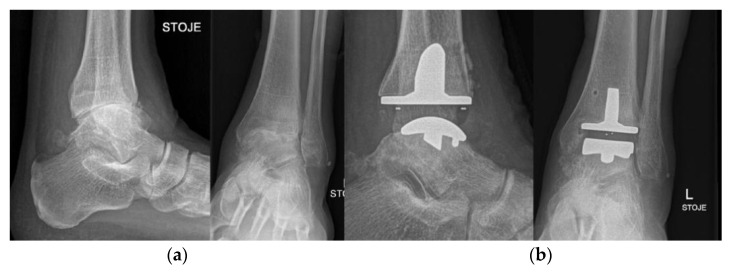
Total ankle arthroplasty (TAA). (**a**) preoperative X-ray; (**b**) postoperative X-ray after total ankle arthroplasty.

With the improvements in implant design and survivorship in the last decade has total ankle arthroplasty (TAA) (Figure 20) again become accepted as an alternative to ankle arthrodesis, which was long considered the gold standard surgical treatment of end-stage ankle osteoarthritis [96]. The procedure is indicated in physically undemanding patients under 60 years of age with an intact joint axis and satisfactory mobility [97]. Multiple arthroplasty techniques and implants have been described for the treatment of first MTP joint osteoarthritis, ranging from Keller resection arthroplasty to interpositional, hemi, and total arthroplasty [50]. MTP arthroplasty began historically with silastic implants and progressed to all-metal implants, whereas recently synthetic cartilage implants have gained popularity [50,98,99].

## 6. Computer-Assisted Orthopaedic Surgery

Computer-assisted orthopaedic surgery (CAOS) includes all kinds of computerized tools, devices, and instrumentations, such as robotic-assisted or clinical navigation technology, but also patient-specific instrumentation and surgical tools that enable an individual, patient-personalized approach, emphasizing safety, and precision in the treatment of musculoskeletal diseases [100,101,102]. There are reports of successful CAOS implementation in orthopaedic surgery from decades ago [103]. Today, this technology has emerged from the laboratory and is being routinely used in operating theatres and might be about to become state-of-the art for certain orthopaedic procedures [102].

CAOS systems use different registration technologies, such as pre-operative image technology, intra-operative medical imaging, and image-free technology, or combinations among them [100]. There are possibilities for using ultrasound computer-assisted orthopaedic surgery systems as an alternative to conventionally used CAOS systems [101]. The active systems may be autonomous, semi-active systems utilizing handheld or controlled forced robotic assisted devices, whereas passive systems only provide guiding information but no direct action [100]. Advances in preoperative imaging and computer planning software pushed the evolution of new techniques such as computer-assisted navigation (CAN), 3D-printed patient-specific instruments (PSI), and robot-assisted surgery, which allow more accurate and reproducible component positioning, and the restoration of joint biomechanics. The development of augmented reality (AR) navigation has focused on improving the safety and efficacy of neurosurgical and orthopedic procedures [104]. With the currently available and expected increase in computational power, it can be expected that AR experiences a geometric increase in applicability in the field of orthopedic surgery [7].

Currently, navigation in spine surgery is primarily used for the pedicle screw placement and evaluation of deformity correction, with the goal of minimizing complications such as infection, excessive blood loss and transfusion, and hardware failure, but this may come at the cost of an increased operative time as registration is a rigorous and time-consuming step in navigation, requiring meticulous soft tissue dissection and bony landmark exposure necessary for accurate point and surface matching [105]. A computer-assisted approach to preoperative pedicle screw placement planning may be another useful tool in spine surgery that can be adapted according to the preferences of the surgeon while integrating the anatomical and structural properties of pedicles and vertebral bodies [106]. Utilizing PSI can be helpful when performing sacroiliac fixation in complex circumstances such as sacral agenesia or when performing an en-bloc surgical resection of sacral chordomas [107,108]. The use of PSI and CAN is also well established in shoulder arthroplasty, where glenoid component positioning continues to pose a challenge, especially for the inexperienced surgeons. Intraoperative navigation technologies have been proved beneficial for optimizing glenoid component placement in both anatomic and reverse TSA [109]. AR, 3D printing, and image-based navigation are modern techniques that may improve implant positioning also in elbow arthroplasty [110]. CT-based 3D preoperative planning can optimize the alignment and may increase survival of total wrist arthroplasty [111]. There are benefits of PSI and electromagnetic navigation use in hip preservation surgery in patients with complex pathoanatomic circumstances, like injury-induced hip dysplasia [112]. PSI, CAN, and robotic-assisted techniques have also proved helpful in knee arthroplasty, where correct frontal, sagittal, and axial alignment of the prosthetic components is essential for the good function and longevity of the implant. New technologies allow more accurate and reproducible restoration of mechanical axis, component alignment, and soft tissue balance [113]. To shorten intraoperative radiation, the 3D-printed personalized guide in assisting the accurate drilling of K-wire has been developed in arthroscopic ankle arthrodesis [114].

These are just a few examples of CAOS concepts implemented in the clinical practice. There is, however, ongoing research on this topic, and the technology keeps on evolving constantly. Thus, CAOS already plays a significant role in the musculoskeletal surgery and will probably even more in the future surgical practice.
